# ESPO and Butler-Williams Symposium: Race, Ethnicity, and Overcoming Barriers to Understand Why Aging Matters

**DOI:** 10.1093/geroni/igaa057.3165

**Published:** 2020-12-16

**Authors:** Roland Thorpe, Jamie Justice

## Abstract

The NIA’s Butler-Williams Scholars Program and GSA’s Emerging Scholars and Professional Organization are united in providing career development opportunities for early career scholars in a manner that promotes leadership, diversity, and inclusivity. This provides a foundation to develop a network of next generation of scientists, clinicians, and policy makers capable of shaping health in aging. Among the chief concerns of our aging population are disparities in health associated with race/ethnicity, experience, sociocultural and socioeconomic factors, as well as access to and communications regarding health care. GSA’s early career professionals and alumni of the prestigious NIA Butler-Williams Scholars Program have tackled these issues directly and the scientific scholarship that results is astounding in its breadth and depth. Dr. Glenna Brewster (Butler-Williams class of 2018) will discuss new findings from a study of African American caregivers of persons living with dementia. Dr. Candace Brown, Ph.D. (Butler-Williams class of 2017), will present on overcoming social and environmental barriers to exercise among older adults. Dr. Joseph Saenz (Butler-Williams class of 2017), will present current work disparities in cognition function in the older Mexican population. The final speaker, Dr. Sarah Forrester (Butler-Williams class of 2019) will explore new perspectives on health equity in physiological dysregulation and aging. In sum, the featured talks by rising stars in aging research deepen our understanding of the influence of race, ethnicity, and overcoming barriers to understand ‘why aging matters’ across our diverse aging populations.

